# High school health education: focus on the development of self-assessment

**DOI:** 10.1186/s12889-026-27180-z

**Published:** 2026-04-09

**Authors:** Barbara Abonyi, József Bognár, István Simon

**Affiliations:** 1 Institute of Sport Science, Eszterházy Károly Catholic University, Eger, Hungary; 2 Sport and Health Science Research Group, Eszterházy Károly Catholic University, Eger, Hungary; 3 Faculty of Pedagogy, Institute of Art and Sport Sciences, University of Sopron Benedek Elek, Sopron, Hungary

**Keywords:** health-education, self-assessment, health-behaviour changes

## Abstract

**Background:**

The present study sought to examine the pedagogical value of developing self-assessment skills through the memorization and regular self-reminding of correct posture among secondary school students. In addition, the research aimed to investigate whether a posture-reminding training method could positively influence self-assessment abilities, health-related behaviors, and stress reduction. The underlying assumption was that beyond the mere provision of theoretical knowledge and instructions related to a healthy lifestyle, it is essential to support students in internalizing and integrating these behaviors into their everyday routines. In the promotion of health-conscious behaviors, self-regulation was considered a key factor alongside formal educational instruction.

**Methods:**

A total of 169 secondary school students (69 boys and 100 girls) from three high schools in Budapest, Hungary, participated in the study (M_age_ = 16.02 ± 0.64). Participants were assigned either to a Habitual Posture Group (HPG) or to a Control Group (CG). The intervention program lasted for seven weeks. Data collection involved questionnaire-based assessments (including the Body Assessment Questionnaire – BAQ), locomotor function tests, and self-report surveys, all administered before and after the intervention period. Statistical analyses were conducted using SPSS software version 25.

**Results:**

Following the intervention, both groups reported increased consumption of fruits, vegetables, and water, as well as a reduction in alcohol intake. The HPG demonstrated greater improvements in engagement with strength-training exercises and in the use of stress management strategies. While significant gains in back muscle strength were observed in both groups, a significant increase in abdominal muscle strength was found only in the HPG at posttest. Interestingly, the CG exhibited more pronounced improvements on the BAQ, which may be attributed to their lower baseline scores.

**Conclusion:**

The findings support the effectiveness of self-assessment as a pedagogical strategy for promoting positive health behavior changes among adolescents. Beyond commonly examined factors such as age, socioeconomic background, and school environment, students’ learning attitudes should also be considered in future psychological and educational research.

## Background

Advancements in medical science and socioeconomic development in industrialized countries have contributed to a continuous increase in average life expectancy. Consequently, there is a growing societal demand not only for longevity but also for sustained well-being, which is closely linked to a disciplined lifestyle characterized by regular physical activity and the reduction of health-compromising behaviors [1, 2]. Promoting health-conscious behavior during childhood and adolescence may yield multiple benefits, including improvements in both immediate and long-term quality of life, as well as the intergenerational transmission of healthier behavioral patterns.

The Health Behavior in School-aged Children (HBSC) study represents a long-standing, internationally recognized initiative aimed at monitoring and promoting health-related behaviors among children and adolescents through a combination of psychological guidance and practical support mechanisms [3]. Hungary has participated in the HBSC program since 1985, enabling the systematic collection and longitudinal analysis of subjective health indicators that contribute to evidence-based public health planning and intervention development.

In 2019, the Hungarian National Center for Public Health launched a comprehensive national initiative targeting health behavior improvement among children and adolescents aged 7–18 years. The program placed strong emphasis on the development of age-specific educational materials and intervention strategies tailored to the lifestyle characteristics and communication preferences of younger generations. Effective health behavior changes typically follow a multistep process that involves identifying harmful habits, increasing awareness, and implementing corrective actions, such as reducing risk factors or promoting the acquisition of health-supportive behaviors. Accordingly, the promotion of healthy lifestyles should extend beyond the mere dissemination of information and focus on facilitating the internalization of health-related behaviors through educational practice and experiential learning [4]. Physical activity habits established during childhood and adolescence are likely to persist into adulthood, underscoring the importance of early intervention. Despite potential structural and motivational barriers, long-term adherence and professional guidance play a crucial role in sustaining health-promoting behaviors. Supporting this perspective, a large-scale international study encompassing 67 countries demonstrated that regular participation in physical education is associated with healthier dietary choices, improved stress resilience, and reduced engagement in harmful behaviors [5].

The HBSC study conceptualizes adolescent health as shaped by social, environmental, and individual determinants operating at multiple levels [6]. In this context, the internalization of health-conscious behaviors during adolescence is facilitated by self-assessment, self-awareness, and reflective self-monitoring at the individual level. Embodied cognition and mind–body approaches further suggest that body awareness and consciously regulated movement enhance self-assessment skills and stress regulation, supporting healthier lifestyle choices [7]. Together, these perspectives provide a theoretical basis for examining posture training as a pedagogical tool for improving body awareness, health behavior, and stress reduction in secondary school students.

Nutrition research consistently identifies daily fruit and vegetable consumption as a key indicator of dietary quality. An average daily intake of at least 400 g, combined with reduced consumption of saturated fats and added sugars and increased intake of lean proteins and dietary fiber, has been shown to lower the risk of chronic non-communicable diseases [8].

Among adolescents, dietary behaviors are strongly influenced by sociocultural factors, including family eating patterns, peer norms, marketing strategies, and the school food environment [9]. The level of health awareness among Hungarian students aged 11–17 years, with daily fruit and vegetable consumption and adequate fluid intake, particularly water and sugar-free beverages, identified as high-priority nutritional behaviors requiring continued public health attention [10].

The development of adolescent risk behaviors is frequently rooted in familial and social modeling processes [11]. Many adolescents who smoke report perceived cognitive and emotional benefits associated with tobacco use, including stress reduction and enhanced concentration [12]. Gender-based analyses consistently indicate that boys are more likely than girls to engage in smoking and alcohol consumption [13]. Although the prevalence of binge drinking among Hungarian youth aged 11–15 years corresponds to national averages, a notable decline has been observed since 2009. Nevertheless, recent trends suggest that girls are gradually narrowing the gender gap, underscoring the need for targeted educational interventions alongside broader policy measures [14].

Adolescents employ a wide range of strategies to cope with stress, with varying degrees of effectiveness. Stress has been identified as a significant risk factor in the initiation of tobacco use during adolescence [15]. Similarly, higher total sugar intake has been reported among students experiencing elevated academic stress, indicating a potential association between stress exposure and maladaptive dietary behaviors [16].

Conversely, physical activity has been consistently shown to exert a protective effect on stress regulation. Adolescents with higher levels of physical activity report lower perceived stress and reduced symptoms of depression, highlighting the role of movement-based interventions in promoting adolescent mental health and resilience [17].

Evidence suggests that current musculoskeletal rehabilitation practices may be insufficient to ensure long-term joint health [18]. Wilmore [19] emphasized that strength development depends not only on muscular adaptations but also on the plasticity of the central nervous system. In this context, the promotion of health-conscious behavior among adolescents requires particular attention to the development of body awareness. Self-assessment can be conceptualized as sustained attentiveness to one’s bodily states, responses, and needs. A range of pedagogical strategies may be employed to foster this form of awareness, including reflective journaling and time-based prompts or reminders, which function both as behavioral regulators and as tools for enhancing self-awareness.

Posture training may play a meaningful role in this process by supporting kinesthetic intelligence and facilitating the development of self-assessment skills. Okunakol et al. reported no significant differences in effectiveness between yoga and Pilates exercises in the management of lumbar spine problems [20]. Similarly, Ramos-Jiménez et al. observed healthier dietary patterns among individuals practicing Hatha yoga compared to non-practitioners [21]. While these effects may partly be attributed to the philosophical foundations of mind–body activities, improvements in body awareness are also likely to contribute to these outcomes. Accordingly, the present study applies a posture training method characterized by slow, controlled, and sustained movements, comparable to those used in mind–body exercise modalities.

Body awareness can be assessed using a variety of measurement tools. In several countries, Postural Assessment Software (PAS/SAPO) is currently undergoing validation for use in both clinical and educational settings [22]. Köteles emphasized the importance of assessment instruments that capture general bodily awareness without focusing on physical symptoms or health complaints [23]. For the purposes of the present study, the Body Awareness Questionnaire (BAQ) was selected due to its conceptual clarity and suitability for adolescent populations. Higher BAQ scores indicate more developed body awareness and self-perception abilities.

Adolescence is a critical period for the development of health-related behaviors and self-regulation, yet secondary school students often have limited access to structured, health-supportive interventions beyond the school setting. As schools represent a key universal context for reducing health inequalities, there is a growing need for low-threshold, pedagogically embedded approaches that foster internalized self-regulation and stress management. Socioeconomic background is a well-established determinant of adolescent health behaviors, including physical inactivity, unhealthy diet, and smoking, underscoring the importance of understanding how socioeconomic status (SES) shapes health education processes and outcomes [24]. Within this context, posture-related self-assessment represents a feasible and scalable strategy that can be integrated into everyday school practice.

The main purpose of this study is to demonstrate the pedagogical value of developing self-assessment skills through the memorization and regular self-reminding of correct posture among secondary school students attending predominantly middle socioeconomic background schools. In addition, the study examines whether posture training contributes to improvements in self-assessment abilities, health-related behaviors, and stress reduction.

## Materials and methods

### Study participants

A total of 169 secondary school students (69 boys and 100 girls) participated in the study. Participants were recruited from three high schools located in Budapest and its surrounding metropolitan area, Hungary. The sampling strategy was applied at the school level, with a primary focus on selecting institutions that predominantly educate students from middle socioeconomic backgrounds and are positioned within the top 25% of the public education ranking. An additional criterion during school selection was the inclusion of both state-funded and non-state-funded schools. Accordingly, one state-funded and two church-funded secondary schools were included in the study.

Each participating school contributed two classes to the study. From each school, the two classes were randomly selected and assigned to either the Habitual Posture Group or the Control Group. Consequently, both research groups comprised classes drawn from different schools, a design intended to increase sample heterogeneity and to minimize potential school-specific bias.

The mean age of the participants was 16.02 ± 0.64 years, and all students were enrolled in full-time secondary education. With regard to prior engagement in posture-related activities, 3.4% of students reported practicing posture exercises daily or two to three times per week, while 5.7% indicated weekly participation. Concerning general physical activity outside school hours, 36.2% of students reported exercising three or more times per week, 45.6% once or twice per week, and 18.2% reported rare or no participation in physical activity.

To ensure group homogeneity, baseline characteristics including frequency of physical activity, prior posture training experience, and mean body fat percentage were taken into account during group allocation (Table 1).

**Table 1:** Characteristics of students’s sample

Groups	*N*	Boys-Girls	Age (M ± SD)	Body fat M %		Physical activity (weekly)	Posture training (weekly)
HPG	94	34 / 60	16.11±0.613	26.22	> 3x	29.8%	3.2%
					1-2x	42.6%	7.4%
					1x / --	27.7%	89.4%
CG	75	35 / 40	15. 93 ± 0.475	19.02	> 3x	36.0%	5.3%
					1-2x	53.3%	8%
					1x /--	10.7%	86.7%

*HPG* Habitual Posture Group, *CG* Control Group

The study was conducted in accordance with the principles of the Declaration of Helsinki [29]. Written informed consent was obtained from the parents or legal guardians of all participants prior to the intervention. Ethical approval was granted by the Institutional Review Board of Eszterházy Károly Catholic University (Approval ID: RK/1243/2021).

### Study design

The intervention was implemented over a seven-week period and followed a pretest–posttest design. Both baseline and post-intervention measurements were conducted. The program integrated theoretical instruction with practical activities related to health behavior and posture awareness.

The initial phase of the intervention focused on reviewing core health-related concepts commonly addressed in Physical Education classes, including the importance of daily physical activity, recommended consumption of fruits, vegetables, and water, and the health impacts of harmful behaviors. In addition, the concept of correct posture was introduced, emphasizing its definition and relevance to musculoskeletal health. Supplementary educational materials, including presentation slides, were made accessible to students via the Microsoft Teams platform.

To ensure consistency across study sites, standardized exercise protocols were developed and distributed to all participating schools. These protocols comprised 14 structured posture-focused training sessions delivered over the seven-week intervention period. Each session lasted approximately 10 min and was integrated into Physical Education lessons following the warm-up phase. Both the Habitual Posture Group (HPG) and the Control Group (CG) participated in weekly reviews of healthy lifestyle criteria. Consequently, members of the CG also received active health-related engagement throughout the study. The primary distinction between the groups was the inclusion of daily and weekly posture-reminding components, the effects of which were the central focus of the investigation.

In addition to the shared program elements, the HPG received targeted instructional support. This included individual consultations, PowerPoint-based educational content, and in-class demonstrations emphasizing correct pelvic alignment, a key component of proper posture and biomechanically efficient movement patterns [25]. Students were presented with examples of both optimal and faulty postures, accompanied by guidance on corrective strategies using strengthening and stretching exercises. Visual aids, including static images and live movement demonstrations, were employed to enhance kinesthetic awareness and to encourage active muscular engagement during movement execution.

To promote continuous self-monitoring of posture, students in the HPG were instructed to set auditory reminders at two-hour intervals using personal mobile devices or classroom-based signals. These reminders functioned as cues for students to perform brief self-assessments and adjust their posture throughout the day, both during school hours and in home settings. In contexts where mobile phone use was restricted, the school bell served as an alternative auditory prompt (Tables 2 and 3).

**Table 2:** Summary of the intervention package

	Pretest	PPT: Healthy lifestyle Correct posture	Healthy lifestyle recall weekly	Posture exercises 2x weekly	Habitual posture training weekly	Posture control to an audible sign in every 2 h	Post-test
HBG	+	+	+	+	+	+	+
CG	+	+	+	+	-	-	+

*HPG* Habitual Posture Group, *CG* Control Group

**Table 3:** Intervention protocol for the habitual posture group

Time	Target	Habitual Posture Training (with tilted pelvis, closed scapulas with the shoulders down)
Week 1	Static positions with correct posture	Standings (closed, normal, straddle, half squat)
Week 2		Sittings on different heights (chair, desk, floor), in different positions (crossed, on the heels, etc.)
Week 3	Dynamical movements with correct posture	Walking of different speeds, jogging, running
Week 4		Arm liftings to different heights Trunk bendings forward, to left/right, trunk rotations
Week 5	Connections of different elements	Sitting down and up to different heights
Week 6		Lifting up and taking down objects
Week 7		Other, individual habitual actings

*HPG* Habitual Posture Group, *CG* Control Group

### Instruments and data collection

Data were collected using a combination of self-report survey instruments, locomotor performance tests, and the Body Awareness Questionnaire – Hungarian version (BAQ-H). Measurements were conducted both prior to (pretest) and following (posttest) the seven-week intervention period.

The survey instrument was designed to assess self-reported musculoskeletal complaints, perceived postural control throughout the day, and a range of health-related behaviors. Based on indicators used in the Health Behavior in School-aged Children (HBSC) reports, the questionnaire included items addressing health-promoting behaviors, such as participation in physical activity outside school hours and daily consumption of raw fruits, vegetables, and water, as well as risk behaviors, including smoking and alcohol consumption. Measuring changes in adolescents’ fluid intake is challenging, as accurate daily recording cannot be reliably expected in this age group. A one-liter threshold was applied in the first survey, however, changes occurring within this range could not be detected. To address this limitation, the second survey specifically asked about perceived increases in fluid intake. As a result, the findings cannot be compared using inferential statistical methods, and the results should be interpreted as indicative rather than fully objective.

In addition, participants were asked to report their stress reduction strategies, such as the use of physical activity, smoking, or alcohol consumption. The survey consisted of closed-ended items yielding nominal (categorical) data.

Locomotor performance tests were employed to assess posture-related muscular endurance. Two static exercises were selected from a 12-item posture control protocol developed by the Hungarian Spine Society [26], which has been applied in previous research. Abdominal muscle endurance was measured using a supine static hold, while back extensor endurance was assessed through a prone position hold. For both tests, the duration (in seconds) for which participants were able to maintain the correct posture was recorded, yielding interval-level data.

Body awareness was assessed using the BAQ-H, which measures individuals’ awareness of internal bodily signals, physiological cycles, and bodily responses. The instrument is based on the concept of a stable internal representation of bodily states and focuses on non-pathological and non-emotional aspects of body awareness [23]. Although the BAQ-H was originally validated in adult populations, the average age of participants in the present study was 16.02 years. Nevertheless, the questionnaire items were considered age-appropriate, clear, and comprehensible for adolescent respondents. Participants rated each item on a 7-point Likert scale, ranging from 1 (strong disagreement) to 7 (strong agreement), with 4 representing a neutral response. This instrument also yielded interval-level data.

### Data analysis

The internal consistency of the questionnaire was evaluated using Cronbach’s alpha, with the Body Awareness Questionnaire – Hungarian version (BAQ-H) demonstrating good reliability (α = 0.855). Descriptive statistics were calculated to summarize the demographic, behavioral, and baseline characteristics of the sample.

Group differences in nominal variables were examined using chi-square tests. Within-group comparisons of pretest and posttest metric data were performed using paired-samples t-tests. For variables that did not meet parametric assumptions or were measured at the nominal level, appropriate nonparametric tests were applied, including the McNemar test for paired categorical data and the Mann–Whitney U test for between-group comparisons. Statistical significance was set at *p* < 0.05 for all analyses. All statistical analyses were conducted using SPSS software version 25.

### Study limitations

Due to logistical and organizational constraints, intact classes were assigned as study groups within each participating school, and students who declined participation were excluded from the study. Consequently, group allocation was conducted at the class level rather than through individual randomization. Two study groups were formed: the Habitual Posture Group (HPG; *n* = 94; 34 males, 60 females) and the Control Group (CG; *n* = 75; 35 males, 40 females). However, due to the active involvement of the CG in health-related educational components of the program, this group did not function as a true control condition. Hence, the study employed a quasi-experimental design, which may limit causal inference and the generalizability of the findings. Another limitation of the study is that accurate recording of exact daily fluid intake cannot be reliably expected in this age group; therefore, a one-liter threshold was applied.

These methodological considerations should be taken into account when interpreting the results.

## Results

### Lifestyle characteristics of the groups

Within the Habitual Posture Group (HPG), 22.3% of students reported consuming fresh fruits and vegetables three to four times per day, 48.9% reported consumption once or twice daily, and 28.7% indicated rare or no consumption. With regard to daily fluid intake, 12.8% of students reported consuming 3–4 L, 50.0% reported 1–2 L, and 37.2% reported consuming 1 L or less.

In terms of health risk behaviors, the majority of students in the HPG (88.3%) reported never having smoked. Alcohol consumption was also relatively low, with 48.9% reporting no alcohol use and 43.6% indicating occasional (rare) consumption. Regarding stress management, 40.4% of students reported having no specific stress-coping strategy. When asked about behavioral changes during the intervention period, 27.7% of students reported an increase in physical activity, while 32.0% reported negative behavioral changes, such as increased smoking or higher consumption of sweets.

In the Control Group (CG), 14.7% of students reported consuming fresh fruits and vegetables three to four times per day, 58.7% reported consumption once or twice daily, and 20.0% reported rare or no consumption. Concerning fluid intake, 18.7% reported daily consumption of 3–4 L, 58.7% consumed 1–2 L, and 22.7% reported consuming 1 L or less.

With respect to smoking behavior, 93.3% of students in the CG reported never having smoked. Alcohol consumption was absent in 56.0% of the group, while 38.7% reported rare alcohol use. Half of the participants in the CG (50.0%) reported not employing any stress-coping method. Regarding behavioral changes during the study period, 35.1% of CG students reported increased physical activity, whereas 14.9% indicated an increase in smoking or sweet consumption (Tables 4 and 5).

**Table 4:** Changes in fruit, vegetable, and fluid intake

	Daily partaking of fruits and vegetables						Daily partaking of water/liquid					
	Before			After			Before			After		
	3-4x	1-2x	Rarely or never	More	Same amount	Less	3–4 L	1–2 L	Less, than 1 L	Optimal amount	Same	Less
HPG	22.3%	48.9%	28.7%	20.2%	69.1%	9.6%	12.8%	50.0%	37.2%	34%	55.3%	9.6%
CG	14.7%	58.7%	26.7%	20%	77.3%	2.7%	18.7%	58.7%	22.7%	46.7%	49.3%	4%

*HPG* Habitual Posture Group, *CG* Control Group

**Table 5:** Changes in risk behavior

	Partaking of alcohol						Smoking					
	Before			After			Before			After		
	Never	Rarely	Frequently	Rarerly	Same	More	Never	Frequently	Daily	Rarerly	Same	More
HPG	48.9%	43.6%	7.4%	33%	16%	2.1%	88.3%	7.4%	4.3%	7.4%	2.1%	2.1%
CG	56%	38.7%	5.3%	28%	14.7%	1.3%	93.3%	1.3%	5.4%	0%	5.3%	1.3%

*HPG* Habitual Posture Group, *CG* Control Group

### Impact of the intervention

Following the intervention, 52% of students in the Habitual Posture Group (HPG) reported that they perceived the program as useful. Statistically significant improvements were observed in several lifestyle-related behaviors within this group. Specifically, participants in the HPG demonstrated a significant increase in the consumption of fresh fruits and vegetables (Wilcoxon Z = − 2.185, *p* = 0.029), as well as a significant increase in daily water intake (Wilcoxon Z = − 5.047, *p* < 0.001). In addition, alcohol consumption decreased significantly following the intervention (Wilcoxon Z = − 2.676, *p* = 0.007). At the time of the second survey, 47.9% of participants in the HPG and 56.0% in the CG indicated that they had not consumed alcohol prior to the intervention; consequently, these participants were excluded from the table. Within the HBG, 28.0% of participants indicated a reduction in alcohol consumption, 14.7% reported no change, while 1.3% reported an increase. No statistically significant changes were observed in smoking behavior or in the use of stress-coping strategies within the HPG.

In the Control Group (CG), 48% of students indicated that they considered their participation in the program useful. Post-intervention analyses revealed a statistically significant increase in daily raw fruit and vegetable consumption (Wilcoxon Z = − 3.381, *p* = 0.001), along with a significant improvement in water intake (Wilcoxon Z = − 4.714, *p* < 0.001). Alcohol consumption also decreased significantly in this group (Wilcoxon Z = − 2.496, *p* = 0.013), with 63.6% of students reporting a reduction, 33.4% reporting no change, and 3.0% reporting increased alcohol use. However, stress-coping behaviors showed a statistically significant but unfavorable shift (Wilcoxon Z = − 2.900, *p* = 0.004), as only a limited number of students adopted physical activity as an alternative to maladaptive coping strategies such as snacking, smoking, or alcohol use. Smoking behavior in the CG remained unchanged following the intervention (Table 6).

**Table 6:** Changes in lifestyle

	HPG	CG	HPG	CG	HPG	CG	HPG	CG	HPG	CG
	cons.of uncooked 2 – cons.of uncooked 1		water intake2 – water intake1		smoking2 - smoking1		alcohol2 - alcohol1		stresscoping2 - stresscoping1	
Z	-2.185	-3.381	-5.047	-4.714	− .447	.000	-2.676	-2.496	− .767	-2.900
A.Sig. (2-tailed)	0.029	0.001	0.000	0.000	0.655	1.000	0.007	0.013	0.443	0.004

*HPG* Habitual Posture Group, *CG* Control Group

Following the intervention, 26.9% of students in the Habitual Posture Group (HPG) reported that they regularly paid attention to their posture. This group demonstrated statistically significant improvements in both locomotor performance tests. Specifically, performance in the back muscle endurance test improved by a mean of 18.25 s (t = − 5.180, df = 92, *p* < 0.001), while abdominal muscle endurance showed a mean improvement of 105.55 s (t = − 3.092, df = 92, *p* = 0.003).

In contrast, only 12.0% of students in the Control Group (CG) reported noticing changes in their posture. Although the CG also exhibited a statistically significant improvement in back muscle endurance, with a mean increase of 25.68 s (t = − 5.734, df = 73, *p* < 0.001, ), no statistically significant improvement was observed in abdominal muscle endurance. Despite a mean increase of 22.47 s, this change did not reach statistical significance (Table 7).

**Table 7:** Changes in posture-related muscular endurance (sec)

			Mean (sec)	SD	df	Differ- ences	CI -95%	CI + 95%	t	Cohen’s d	Sig. (2-tailed)
back muscles	HPG	pretest	96.322	41.227	4.2750	-11.254	87.71	104.5	-5.734	0.387	0.000
		posttest	114.575	51.037	5.292		104.1	125.1			
	CG	pretest	92.797	48.627	5.652	-16.750	81.53	104.1	-5.180		0.000
		posttest	118.473	63.115	7.336		103.9	133.1			
abdo.muscles	HPG	pretest	261.849	196.607	20.387	-37.753	220.2	300.5	-1.330	0.194	0.003
		posttest	367.397	381.239	39.532		288.9	445.9			
	CG	pretest	330.226	202.593	23.393	11.194	283.6	376.8	-3.092		0.188
		posttest	352.693	210.526	24.309		304.3	401.1			

*HPG* Habitual Posture Group, *CG* Control Group

In the Habitual Posture Group (HPG), the mean Body Awareness Questionnaire (BAQ) score increased from 74.34 ± 14.19 at pretest to 75.63 ± 13.94 at posttest, representing a mean improvement of 1.29 points. In contrast, the Control Group (CG) demonstrated an increase in BAQ scores from 71.00 ± 15.49 at pretest to 73.90 ± 15.15 at posttest, corresponding to a mean improvement of 2.90 points in self-assessment (Table 8).

**Table 8:** Changes in BAQ-H scores

Type	*N*	Subset for alpha = 0.05	
		1	
		BAQ-H 1	BAQ-H 2
Habitual Posture Group	94	74.3404	75. 6344
Control Group	75	71.0000	73.9067

## Discussion

A wide range of health behavior development programs targeting children and adolescents have focused primarily on discrete lifestyle components such as hydration, nutrition, or the reduction of health-compromising behaviors. The present study extends this approach by embedding body awareness and self-assessment as core pedagogical mechanisms within a comprehensive health behavior intervention aligned with the HBSC conceptual model, which conceptualizes health as shaped by interacting individual, social, and environmental determinants [4, 5]. Implemented in secondary schools predominantly educating students from middle socioeconomic backgrounds the intervention shifted the emphasis from information transmission toward reflective self-monitoring and posture-related awareness, aiming to support the internalization and regulation of health-related behaviors rather than their short-term adoption.

Both study groups exhibited favorable changes in selected lifestyle indicators, including increased fruit and vegetable consumption, improved water intake, and reduced alcohol consumption. These results corroborate earlier school-based health promotion studies demonstrating that relatively brief interventions can produce measurable improvements in adolescent dietary behaviors [27]. However, a more nuanced analysis revealed important differences between the groups. While the Control Group (CG) showed domain-specific improvements in nutrition and hydration, these gains coincided with a slight deterioration in stress-coping strategies, suggesting that behavior change confined to isolated domains may lack stability and does not necessarily generalize across broader dimensions of health behavior. In contrast, the Habitual Posture Group (HPG) demonstrated concurrent improvements across multiple lifestyle indicators without a parallel decline in coping behaviors, indicating a more integrated and sustainable pattern of change.

The observation that a higher proportion of CG participants subjectively reported overall lifestyle improvement, despite less consistent objective change, warrants careful interpretation. This apparent discrepancy is best understood in relation to baseline group differences within a relatively homogeneous, middle socioeconomic school context. The HPG comprised predominantly students with higher initial levels of health-conscious behavior, stronger intrinsic motivation, and greater pedagogical support from Physical Education teachers. Approximately two-thirds of the HPG were female, and their baseline profiles reflected more advanced health behavior patterns. Consequently, improvements in the CG may have been perceived as more salient due to lower starting levels, whereas objectively meaningful changes in the HPG may have been less noticeable to participants themselves.

These findings align with those of Sharma et al. (2018), who reported improvements in dietary and alcohol-related behaviors following a short-term, curriculum-integrated school intervention. The absence of significant changes in smoking behavior across both groups likely reflects a floor effect, as smoking prevalence was already low at baseline. Additionally, self-reported measures of sensitive behaviors are inherently susceptible to social desirability bias, particularly in school-based settings, where adolescents may underreport behaviors perceived as socially undesirable. This limitation should be considered when interpreting null findings in this domain [28].

However, whereas Sharma et al’s outcomes were largely limited to explicitly targeted behaviors, the present results suggest that interventions incorporating self-monitoring and embodied awareness may facilitate broader lifestyle regulation. This distinction highlights the added pedagogical value of integrating body-based self-assessment strategies into health promotion efforts, even within relatively advantaged school environments.

Marked group differences were observed in posture-related outcomes and locomotor performance. CG participants reported limited engagement in posture self-monitoring, consistent with the absence of targeted self-awareness strategies. Although they received posture-related instruction and exercises, their engagement remained situational and externally guided, resulting in improvements restricted to back muscle endurance. In contrast, the HPG was systematically encouraged to perform repeated posture self-checks throughout the day, emphasizing pelvic control through abdominal and gluteal activation. This approach was associated with significant improvements in both back and abdominal muscle endurance. Importantly, lower baseline values in these measures may have provided greater scope for improvement, underscoring the need to account for initial performance levels when evaluating intervention effects.

Overall, the results are consistent with the HBSC approach to adolescent health, which conceptualizes health behavior as shaped by interacting individual characteristics and broader social and environmental contexts. From a mind–body and embodied cognition perspective, the posture-focused intervention appears to have enhanced body awareness and stress regulation, thereby supporting healthier lifestyle choices.

Both groups showed improvements in Body Awareness Questionnaire (BAQ-H) scores, indicating that self-assessment skills are amenable to development during adolescence. However, higher baseline scores in the HPG likely constrained the magnitude of observable change, consistent with a ceiling effect [27]. As a result, the true impact of the posture-reminding intervention on self-assessment abilities in this group may be underestimated. The use of repeated prompts aligns with established behavior change techniques emphasizing self-monitoring as a key driver of sustained behavioral regulation [28].

## Conclusions

The findings of the present study suggest that school-based health behavior interventions that incorporate body awareness and posture-related self-monitoring may promote an integrated pattern of lifestyle change among adolescents. Although both the Habitual Posture Group (HPG) and the Control Group (CG) demonstrated improvements in selected lifestyle indicators such as fruit and vegetable consumption, daily water intake, and alcohol use, the HPG showed a more coherent pattern of behavioral adaptation. This included improvements in posture-related muscular endurance without the unfavorable shift in stress-coping behaviors observed in the CG.

These results indicate that pedagogical approaches emphasizing repeated posture self-checks and reflective self-monitoring may strengthen adolescents’ body awareness and support the regulation of health-related behaviors. Consistent with the HBSC perspective, which conceptualizes adolescent health as shaped by interacting behavioral and contextual factors, the posture-focused intervention appears to facilitate a more coherent process of lifestyle regulation.

From an educational perspective, the incorporation of simple and scalable tools such as posture reminders into school-based physical education or health promotion programs may provide a feasible strategy for fostering sustainable health behavior development during adolescence. Future research should examine the long-term sustainability of these effects and test the intervention in larger and more socioeconomically diverse school populations using longitudinal designs and objective behavioral measures.

## Data Availability

The surveys that support the findings of this study are available from the Sports and Health Research Group, Eger. The data will be available and transparent.
